# Spinal PKCα inhibition and gene-silencing for pain relief: AMPAR trafficking at the synapses between primary afferents and sensory interneurons

**DOI:** 10.1038/s41598-018-28512-9

**Published:** 2018-07-06

**Authors:** Olga Kopach, Volodymyr Krotov, Angela Shysh, Andrij Sotnic, Viacheslav Viatchenko-Karpinski, Victor Dosenko, Nana Voitenko

**Affiliations:** 1 Department of Sensory Signalling, Bogomoletz Institute of Physiology, Kyiv, Ukraine; 2 Department of Clinical and Experimental Epilepsy, Institute of Neurology, University College London, London, UK; 3 Department of General and Molecular Pathophysiology, Bogomoletz Institute of Physiology, Kyiv, Ukraine; 4 Kyiv Academic University, Kyiv, Ukraine; 5 Present Address: The University of Alabama at Birmingham, Birmingham, United States

## Abstract

Upregulation of Ca^2+^-permeable AMPA receptors (CP-AMPARs) in dorsal horn (DH) neurons has been causally linked to persistent inflammatory pain. This upregulation, demonstrated for both synaptic and extrasynaptic AMPARs, depends on the protein kinase C alpha (PKCα) activation; hence, spinal PKC inhibition has alleviated peripheral nociceptive hypersensitivity. However, whether targeting the spinal PKCα would alleviate both pain development and maintenance has not been explored yet (essential to pharmacological translation). Similarly, if it could balance the upregulated postsynaptic CP-AMPARs also remains unknown. Here, we utilized pharmacological and genetic inhibition of spinal PKCα in various schemes of pain treatment in an animal model of long-lasting peripheral inflammation. Pharmacological inhibition (pre- or post-treatment) reduced the peripheral nociceptive hypersensitivity and accompanying locomotive deficit and anxiety in rats with induced inflammation. These effects were dose-dependent and observed for both pain development and maintenance. Gene-therapy (knockdown of PKCα) was also found to relieve inflammatory pain when applied as pre- or post-treatment. Moreover, the revealed therapeutic effects were accompanied with the declined upregulation of CP-AMPARs at the DH synapses between primary afferents and sensory interneurons. Our results provide a new focus on the mechanism-based pain treatment through interference with molecular mechanisms of AMPAR trafficking in central pain pathways.

## Introduction

Targeting the mechanisms underlying impairments for disease treatment is a concept of precision medicine for emerging advanced therapeutics with limited side effects. Over decades of exploring the mechanisms of pain development and maintenance for effective interception of persistent pain at the cellular and molecular levels, glutamate AMPA receptors (AMPAR) are of a particular interest among various systems being outlined. This is due to the principal role of AMPARs in fast transmission and because of AMPAR upregulation causing central sensitization of the dorsal horn (DH), a specific form of spinal plasticity mediating pain^1–3^. Changes in the activity-dependent trafficking of AMPARs have been well documented in the DH interneurons in chronic pain conditions where two mechanisms take place: internalization of GluA2-containing, Ca^2+^-impermeable AMPARs away from the synapses between primary afferents and DH interneurons^4–6^, while insertion of GluA1-containing, Ca^2+^-permeable (CP) AMPARs into the extrasynaptic sites^7,8^. Either mechanism increases the AMPAR-mediated Ca^2+^-conductance in sensory interneurons, triggering the long lasting hyperexcitability of central nociceptors^9^ and, hence, Ca^2+^-dependent pain chronification^2^. Our recent study has demonstrated that spinal administration of the activity-dependent antagonists of CP-AMPARs had alleviated inflammatory pain hypersensitivity at the periphery without any detectable side effects that further supports the role of spinal CP-AMPARs in chronic pain^10^.

Predominantly, upregulation of CP-AMPARs depends on the protein kinase C alpha (PKCα) activation. This molecular mechanism includes phosphorylation of GluA2 subunit by PKCα that disrupts binding of the receptor to its synaptic anchoring protein, causing, thereby, the receptor’s internalization away from the postsynaptic density^5^. Our previous studies demonstrated that PKC antagonists^5,10^ or genetic targeting the PKCα^8^ alleviated the inflammatory-induced nociceptive hypersensitivity. Though it provides a proof-of-concept of targeting the spinal PKCα for pain relief, there has been, however, no systematic exploration of the therapeutic profile of such targeting to addressing both development and maintenance of persistent pain (essential for preclinical translation). Furthermore, whether PKCα inhibition has an effect on balancing the inflammation-induced upregulation of CP-AMPARs at the synapses between primary afferents and nociceptive interneurons also remains unknown.

In this study, we combined behavioural testing *in vivo* with electrophysiology in the spinal cord *in situ* to determine the therapeutic potential of targeting the spinal PKCα in relieving persistent inflammatory pain through pharmacological inhibition or a gene-silencing approach (knockdown). Gene targeting holds great promise as a therapy that could feasibly replace repeatedly injected drugs, gaining an ever-increasing interest for its therapeutic potential in persistent pain treatment. Moreover, the gene-silencing approach does not involve permanent gene modification and, thereby, could provide long-lasting, still reversible, effects on neurons. Therefore, one of our goals was to examine the effect of different experimental schemes for gene-therapy: pre- and post-treatment of persistent inflammatory pain in an animal model of long-lasting peripheral inflammation. The outcome of either pharmacological inhibition or gene-silencing approach on the inflammation-induced upregulation of CP-AMPARs at the DH synapses was also demonstrated through recordings of the AMPAR-mediated postsynaptic currents evoked with primary afferent (dorsal root) stimulation in the superficial DH interneurons.

## Results

For targeting the spinal PKCα we have utilized two different approaches: pharmacological inhibition and gene silencing through local delivery of a drug into the lumbar spinal cord. The CFA-induced model of long lasting peripheral inflammation was used to produce persistent nociceptive hypersensitivity in rats, which could persist for over several weeks after the induction of inflammation if left untreated^5,8,10^. Different experimental schemes (pre- or post-treatment) were probed to figure out the therapeutic effects of spinal PKCα inhibition on the developing inflammatory pain and its maintenance. Next, the effects on locomotive deficit and anxiety of animals with peripheral inflammation were determined. Ultimately, the postsynaptic AMPAR-mediated currents were recorded in DH interneurons to assess whether PKCα inhibition arrested the inflammatory upregulation of CP-AMPARs at the DH synapses between primary afferents and sensory interneurons.

### Spinal PKC inhibition declines the development of both nociceptive hypersensitivity and locomotive deficit in animals with induced peripheral inflammation

**Figure 1:**
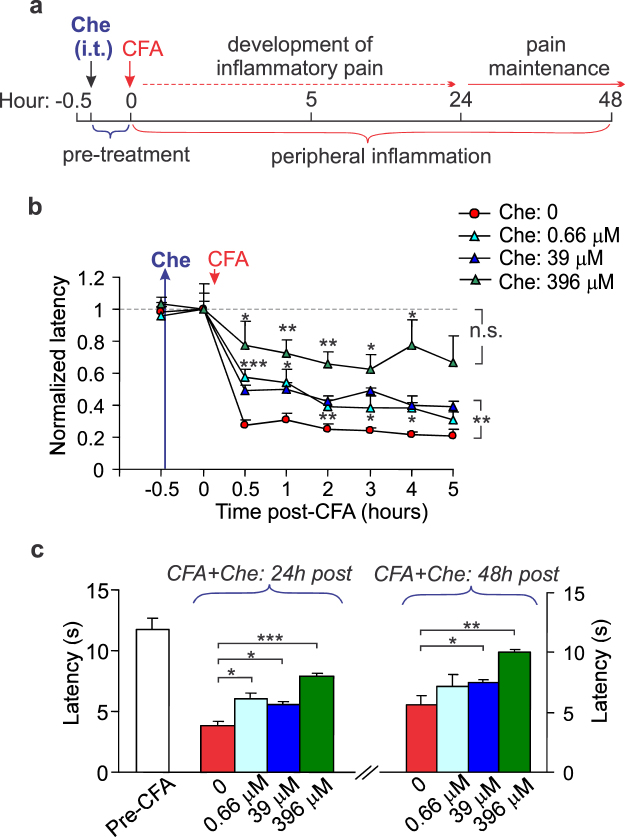
Pharmacological inhibition of spinal PKC alleviates the peripheral nociceptive hypersensitivity in rats. (**a**) A cartoon depicting experimental scheme of pre-treatment with chelerythrine (Che, 10 μl/rat), given intrathecally (i.t.) prior to the induction of peripheral inflammation with an intraplantar injection of CFA (30 min in advance). (**b**) The time course of changes in the thermal nociceptive threshold following i.t. administration of chelerythrine at different concentrations. The threshold (the paw withdrawal latency) normalized to the time of CFA injection (“0” time; induction of inflammation). (**c**) Summary of the nociceptive threshold (the paw withdrawal latency) in CFA-inflamed animals on the days 1 and 2 post-CFA after pre-treatment with chelerythrine at different concentrations. All data are shown as mean ± SEM. *P < 0.05, **p < 0.01, ***p < 0.001, n.s., non-significant *vs.* the corresponding time-point in the CFA-inflamed group without chelerythrine.

For PKC inhibition, a potent, cell-permeable PKC inhibitor, chelerythrine, was initially used. In untreated animals (no pre-treatment with chelerythrine), an intraplantar injection of CFA produced a robust peripheral thermal hypersensitivity, which could be detected on the ipsilateral hindpaw within as short time period as half an hour, being severely developed further (Fig. 1b). Chelerythrine given shortly prior to the induction of peripheral inflammation (30 min in advance; Fig. 1a) alleviated the thermal hypersensitivity with a high potency. We detected the antinociceptive effect of chelerythrine at 660 nM (IC_50_ for PKC inhibition), which emerged rapidly (within 30 min after i.t. injection) as a decline in the CFA-induced drop of the nociceptive threshold on the ipsilateral (inflamed) hindpaw (the threshold increased by ~168%, *n* = 5 rats/group, *p* < 0.001 compared with the corresponding time-point in the inflamed group without chelerythrine; Fig. 1b). The effect was time-dependent and weakened following the next 2 days (Fig. 1c). Increasing the concentration of chelerythrine resulted in the antinociceptive effect of longer duration. At 39 μM, chelerythrine produced an approximate two-fold rise in the thermal threshold (n = 5 rats/group, *p* < 0.01 compared with the CFA-inflamed group without chelerythrine; Fig. 1b). The effect remained for the next 2 days (increase in the threshold by ~46% and ~41%, *p* < 0.05 on the days 1 and 2, respectively; Fig. 1c). Chelerythrine at 396 μM completely abolished the peripheral nociceptive hypersensitivity that the threshold in inflamed rats at 5 h post-CFA became reversing to control level (before inflammation, *n* = 4 rats/group, *p* > 0.2 paired comparison; Fig. 1b). The effect remained steady when re-determined on the days 1 and 2 post-CFA (increase in the threshold by ~105%, *p* < 0.001 and ~81%, *p* < 0.01, respectively, compared with the corresponding latency in CFA-inflamed group without chelerythrine; Fig. 1c).

We probed the time-dependence of the antinociceptive effects produced by chelerythrine through modifying the experimental scheme of pre-treatment by extending the time of a drug administration up to 3 h in advance (Supplementary Fig. S1a). However, the antinociceptive effect was minor, if any, for both micromolar and millimolar concentrations of chelerythrine (*p* > 0.05; Supplementary Fig. S1b). Some effect was observed for higher chelerythrine concentrations on the day 1 post-CFA (the thermal threshold increased by ~47%, n = 8, *p* < 0.05 for 39 μM chelerythrine and by ~36%, n = 3, *p* < 0.05 for 3.96 mM chelerythrine compared with the inflamed group without chelerythrine; Supplementary Fig. S1c).

**Figure 2:**
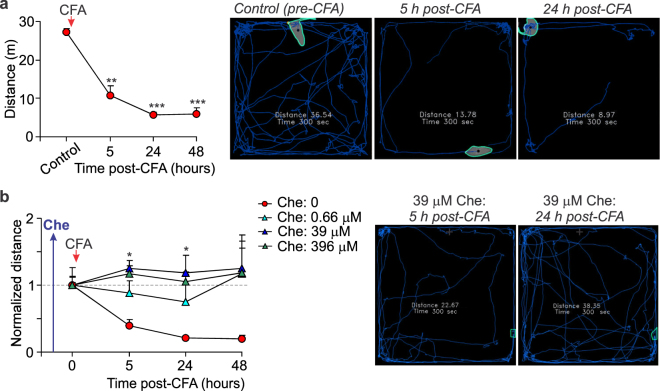
Spinal PKC inhibition declines locomotive deficit in animals with induced peripheral inflammation. (**a**) Summary of the total distance travelled by animals before and after the induction of CFA-induced peripheral inflammation (left graph) and representative snapshots of the analysed open-field test recordings (taken from the same animal over the time of testing) depicting animal’s movement for the 5-min duration (images on the right). **P < 0.01, ***p < 0.001 *vs*. control (before CFA injection); n = 9 animals/group. (**b**) Summary of the average distance travelled by CFA-inflamed animals those received pre-treatment with chelerythrine at different concentrations (left graph) and representative snapshots of the analysed open-field test recordings taken from a CFA-inflamed animal after pre-treatment with chelerythrine (39 μM, treatment scheme as shown in Fig. 1a). All data are shown as mean ± SEM. *P < 0.05 *vs.* the corresponding time-point in the CFA-inflamed group without chelerythrine.

Peripheral nociceptive hypersensitivity was associated with a robust locomotive deficit that inflamed animals exhibited rapidly after the induction of inflammation (within an hour), which could persist for over days (Fig. 2a). Inhibition of spinal PKC with chelerythrine given 30 min before the induction of peripheral inflammation abolished the development of locomotive deficit in rats after CFA injection. The total distance of animal movements within the open-field arena was similar to control (“0” time, pre-inflammatory level) over the time-course of testing for all groups of CFA-inflamed animals treated with different concentrations of chelerythrine (*p* > 0.1; Fig. 2b).

Animals with locomotive deficit often exhibited the anxiety-like behaviour. Consistent with our previous studies^10,11^, animals with peripheral inflammation and pain hypersensitivity typically sat within the arena corners, preferably not moving across the open area of the arena (Fig. 2a). However, neither signs of anxiety were observed in animals with peripheral inflammation those received pre-treatment with chelerythrine at different concentrations – animals displayed a common exploratory behaviour pattern by moving freely across the open-field arena and through the centre (Fig. 2c).

### Spinal PKC inhibition shortens the pain maintenance and recovers both locomotive deficit and anxiety of animals with peripheral inflammation: chelerythrine and PKCα (C2-4) inhibitor peptide

**Figure 3:**
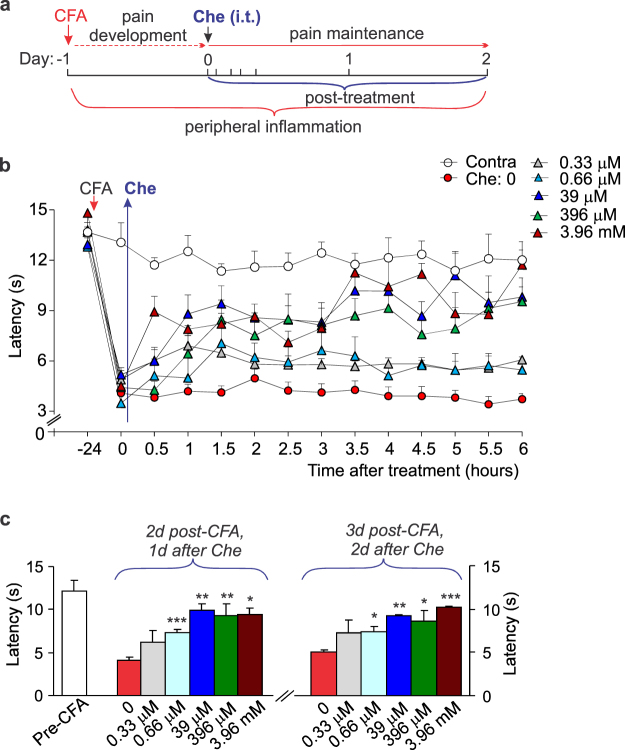
Spinal PKC inhibition shortens the maintenance of persistent inflammatory pain in rats. (**a**) A cartoon depicting experimental scheme of post-treatment of the CFA-induced peripheral hypersensitivity with chelerythrine (Che, intrathecal, i.t., administration, 10 μl/rat) for the assessment of inflammatory pain maintenance. (**b**) The time course of changes in the thermal nociceptive threshold (the paw withdrawal latency) in CFA-inflamed rats treated with chelerythrine at different concentrations (“0” is the time of chelerythrine injection, a start of treatment). (**c**) Summary of the thermal nociceptive threshold in CFA-inflamed rats on the days 1 and 2 following post-treatment with chelerythrine at different concentrations. All data are mean ± SEM. *P < 0.05, **p < 0.01, ***p < 0.001 *vs*. the CFA-inflamed group without chelerythrine.

Thus far, the observed effects of spinal PKC inhibition demonstrate the therapeutic potential of such a targeting in pain treatment and against animal anxiety when applied shortly in advance before the induction of inflammation. However, therapy implemented as a post-treatment is more preferable towards clinical relevance. Therefore, we next examined the therapeutic effects of spinal PKC inhibition used as a post-treatment of persistent inflammatory pain. Post-treatments were initiated 1 d post-CFA (Fig. 3a), the time-point representing the inflammatory pain maintenance^5,10,12^.

Chelerythrine markedly alleviated the thermal nociceptive hypersensitivity following its post-treatment administration, with a high efficiency. The antinociceptive effect was observed following i.t. injection of chelerythrine at the concentration of 660 nM (IC_50_ for PKC inhibition), which appeared shortly (within 2 h, an increase in the threshold was by ~25%, n = 5, *p* < 0.05; Fig. 3b). The antinociceptive effect further augmented with increasing the chelerythrine concentration – the effect has been also longer persisted. At 39 μM of chelerythrine, an increase in the thermal latency has been detected within 30 min (by ~57%, n = 12, *p* < 0.05) and gradually raised up (by ~110% at 1 h and by ~128% at 1.5 h, *p* < 0.01 compared with the corresponding time-point in CFA-inflamed animals without chelerythrine; Fig. 3b); also, it remained steady for over the next days (Fig. 3c).

**Figure 4:**
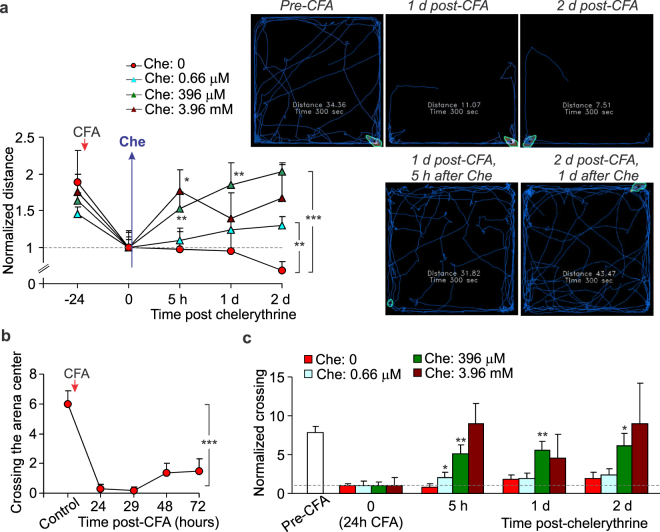
Pharmacological inhibition of spinal PKC with chelerythrine restores both locomotive deficit and anxiety of animals with peripheral inflammation. (**a**) Summary of the total distance travelled by CFA-inflamed animals before and following post-treatment with chelerythrine at different concentrations (left graph) and representative snapshots of the analysed open-field test recordings taken from the same animal at different time-points of testing. Upper row: images depicting movements of a CFA-inflamed animal with no chelerythrine treatment. Lower row: images of a CFA-inflamed animal following post-treatment with 396 μM chelerythrine. Images are taken from the same rats over the time of testing. The average distance normalized to the time of chelerythrine administration (“0” time, a start of treatment); experimental scheme for post-treatment was as in Fig. 3a. (**b**) The time course of changes in animal explorative behaviour as readout of anxiety produced by persistent peripheral inflammation. (**c**) Summary of changes in animal explorative behaviour following post-treatment with chelerythrine at different concentrations. The number of crossing the arena centre normalized to chelerythrine administration (“0” time that is 24 h post-CFA). All data are mean ± SEM. *P < 0.05, **p < 0.01, ***p < 0.001 *vs*. the corresponding time-point in CFA-inflamed group without chelerythrine or *vs*. control (before CFA injection) for Fig. b.

In addition to the antinociceptive effect, chelerythrine markedly reduced the locomotive deficit in animals with persistent peripheral inflammation. Such an effect, being rapidly emerging (within hours), was concentration-dependent and progressive (Fig. 4a). At higher concentrations, chelerythrine recovered impaired locomotion of CFA-inflamed animals back to control (pre-inflammatory) level. In particular, the mean distance travelled by rats was: 24.6 ± 1.7 m in control (non-inflamed group) but 15 ± 1.5 m in the inflamed animals 1 d post-CFA and 22.9 ± 3.5 m in the inflamed, Che-treated animals at 1 d post-CFA, 5 h after 396 μM chelerythrine (n = 6 /group). Chelerythrine produced a decrease in anxiety of animals with persistent peripheral inflammation (Fig. 4b,c). Animals with peripheral inflammation showed recovery in their explorative behaviour following treatment with chelerythrine at different concentrations – inflamed animals increasingly crossed the open-field arena by freely entering the arena centre (Fig. 4c) that reflects a lack of anxiety.

**Figure 5:**
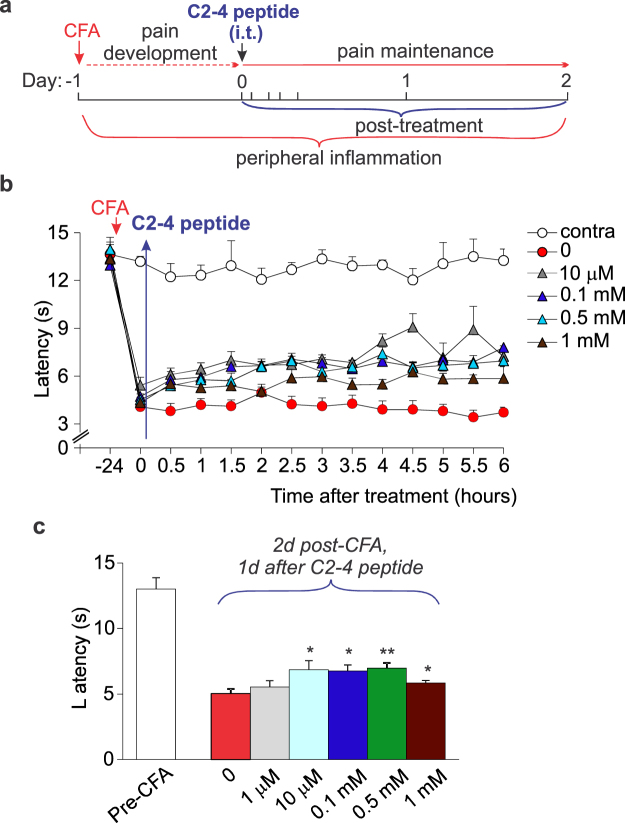
The antinociceptive effect of PKCα (C2-4) inhibitor peptide in rats with induced peripheral inflammation. (**a**) A cartoon depicting experimental scheme of post-treatment of the CFA-induced peripheral hypersensitivity with PKCα (C2-4) inhibitor peptide (intrathecal administration, i.t., 10 μl/rat). (**b**) The time course of changes in the thermal nociceptive threshold (the paw withdrawal latency) in the CFA-inflamed rats treated with C2-4 peptide at different concentrations. “0” time is 24 h post-CFA when treatment had been initiated. (**c**) Summary of the thermal nociceptive threshold in CFA-inflamed rats on the day 2 post-CFA after treatment with C2-4 peptide at different concentrations (that is the day 1 after initiating post-treatment). All data are mean ± SEM. *P < 0.05, **p < 0.01 *vs.* the CFA-inflamed group without C2-4 peptide.

For the selective pharmacological inhibition of PKCα subtype, the antinociceptive effects of PKCα (C2-4) inhibitor peptide were also examined, which use has not been yet reported *in vivo*. Although there was no clear effect of C2-4 peptide administered at the concentration of 1 μM (n = 4, *p* > 0.05), the peptide at 10 μM concentration produced a significant increase in the nociceptive threshold in CFA-inflamed rats, which was appeared within half an hour (*p* < 0.05; Fig. 5a) and remained on the days 1 and 2 post-CFA (increase in the threshold by ~26% and ~17%, respectively, *p* < 0.05; Fig. 5b). Increasing the C2-4 peptide concentration from 100 μM (n = 5) to 1 mM (n = 4) produced further increase in the threshold (by ~50% to 60%, *p* < 0.05 for each concentration tested; Fig. 5b), resulting in pain alleviation observed within 0.5-1 h after i.t. administration and persisted for over few days (*p* < 0.05; Fig. 5c).

### Gene silencing of spinal PKCα for antinociception

**Figure 6:**
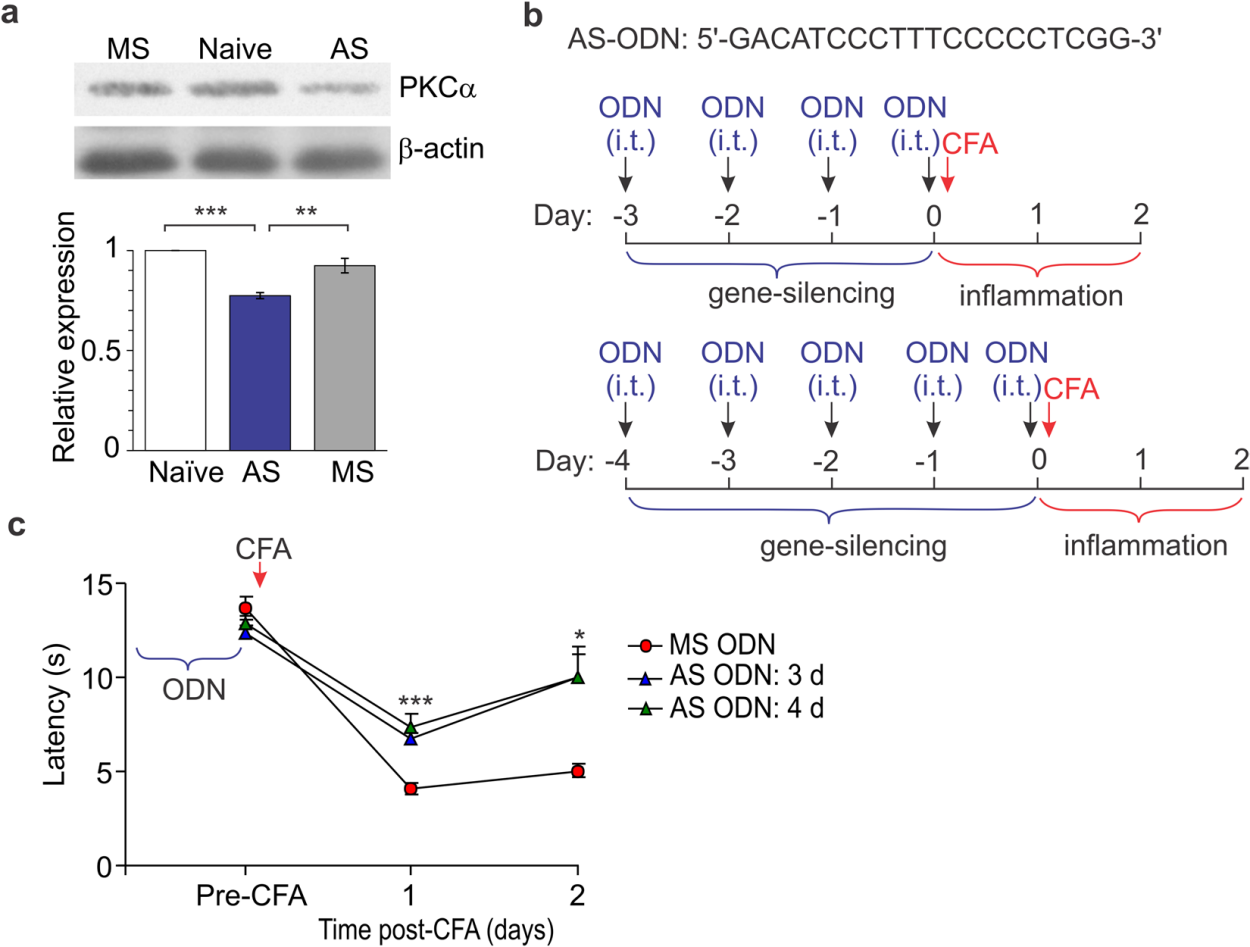
Gene-silencing of spinal PKCα reduces the inflammatory pain maintenance. (**a**) Western blot analysis of the PKCα protein expression level in the dorsal horn of spinal cord in rats after intrathecal injections of AS ODN specific to PKCα, but not MS ODN. Top: Western blot examples for PKCα protein (80 kDa) and beta-actin (43 kDa). The examples are original images taken using the same exposition. Bottom: Summary of the PKCα protein expression level in experimental groups (n = 6 rats/group) relative to naïve. The PKCα protein expression was normalized to the corresponding beta-actin. (**b**) Cartoons of the experimental schemes of gene-therapy (gene-silencing of spinal PKCα) with AS ODN of the indicated sequence (upper row) administered intrathecally (i.t., 10 μl/rat). (**c**) The time-course of changes in the thermal nociceptive threshold (the paw withdrawal latency) in rats with persistent peripheral inflammation those received gene therapy with oligodeoxynucleotides (AS ODN, MS ODN) in accord with the experimental schemes as above. All data are mean ± SEM. *P < 0.05, **p < 0.01, ***p < 0.001* vs.* the corresponding time-point in the CFAinflamed group treated with MS ODN or as indicated in (**a**).

To ultimately determine whether genetic inhibition of spinal PKCα would produce a marked and lasting alleviation of inflammatory pain at the periphery, the gene therapy – gene silencing with AS ODN specific to PKCα – was next implemented. The reduced expression of PKCα protein at the DH of the spinal cord after i.t. administration of AS ODN, but not MS ODN, was confirmed with Western blot analysis (by ~23%, n = 6 rats/group, *p* < 0.01; Fig. 6a), fully consistent with our previous observations^8^. Gene silencing of the DH PKCα produced a reduction in the thermal nociceptive hypersensitivity of inflamed rats when applied as either pre- or post-treatment of the CFA-induced inflammatory pain. Gene-therapy initiated before the induction of peripheral inflammation resulted in a profound antinociception; its therapeutic effect, nevertheless, was  not strictly determined by duration of the therapy (Fig. 6b,c). After daily i.t. administration of AS ODN for 3 days, the thermal nociceptive threshold in CFA-inflamed rats increased by ~64% (n = 8, *p* < 0.001) and by ~70% (*p* < 0.05 compared with the MS ODN-treated group) on the days 1 and 2 post-CFA, respectively (Fig. 6c). Such administration of AS ODN for 4 days produced the increase by ~84% (n = 6, *p* < 0.001) and ~98% (*p* < 0.05), respectively (Fig. 6c).

**Figure 7:**
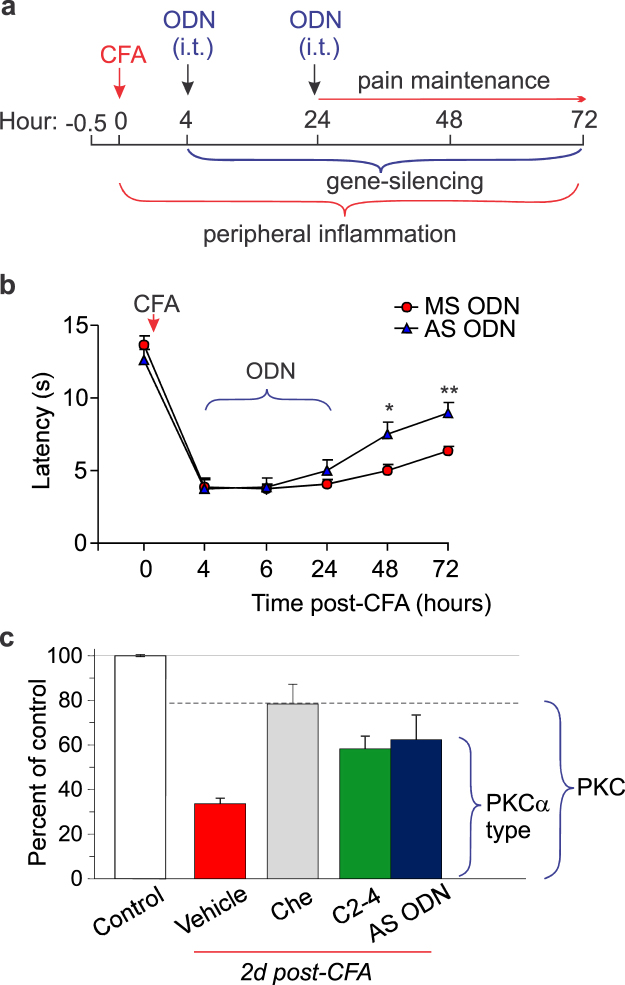
Gene-therapy produces alleviation in the persistent inflammatory pain. (**a**) A cartoon of the experimental scheme of gene-therapy with oligodeoxynucleotides specific to PKCα administered intrathecally (i.t., 10 μl/rat) after the peripheral nociceptive hypersensitivity had developed. (**b**) The time-course of changes in the thermal nociceptive threshold (the paw withdrawal latency) in the CFA inflamed rats those received treatment with oligodeoxynucleotides (AS ODN, MS ODN) as indicated above. (**c**) Summary of changes in the thermal nociceptive threshold in control and CFA-inflamed groups those received different post-treatment for estimation of the spinal PKCα contribution into the peripheral inflammatory pain maintenance. The relative thermal threshold is  the maximum efficiency for each antagonist tested. Control is non-inflamed animals. All data are mean ± SEM. *P < 0.05, **p < 0.001 *vs.* the corresponding time-point in the CFA-inflamed group treated with MS ODN.

Gene-therapy initiated as a post-treatment of persistent inflammatory pain (Fig. 7a) also alleviated the thermal nociceptive hypersensitivity at the periphery. Initiation of the therapy shortly after the induction of inflammation (4 h post-CFA) resulted in the increased thermal latency in rats with peripheral inflammation by ~48% (n = 8, *p* < 0.05) on the day 2 post-CFA and by ~43% (*p* < 0.01 compared with the MS ODN-treated group) on the day 3 (Fig. 7b).

### PKCα-dependent inflammatory upregulation of CP-AMPARs in the DH synapses between primary afferents and sensory interneurons: pharmacological inhibition and gene silencing

**Figure 8:**
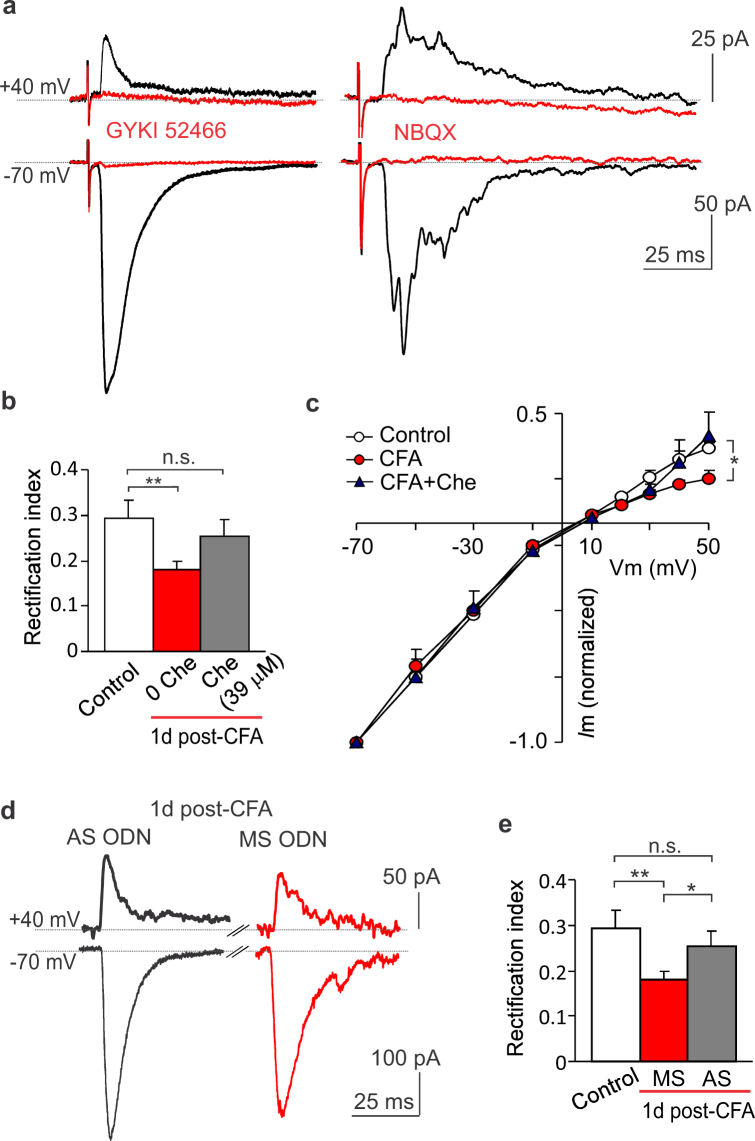
Targeting the spinal PKCα results in ablating the upregulated CP-AMPARs at the DH synapses between primary afferents and sensory interneurons in persistent inflammatory conditions. (**a**) Examples of the AMPAR-mediated excitatory postsynaptic currents (EPSCs) evoked by primary afferent stimulation in DH interneurons recorded at different membrane potentials. The AMPAR blockers GYKI 52466 and NBQX eliminated the currents at –70 mV and +40 mV (red traces). (**b**,**c**) Summary of the rectification index of the AMPAR-mediated EPSCs (**b**) and their *I-V* relationship (**c**) in DH neurons from control (no inflammation), the CFA-inflamed, non-treated and the CFA-inflamed animals  given 39 μM chelerythrine (experimental scheme as in Fig. 3a). The rectification index was calculated as the ratio of the current amplitude at +40 mV to that at –70 mV. (**d**) Examples of the AMPAR-mediated EPSCs recorded at –70 mV and +40 mV in DH interneurons from the CFA-inflamed groups after gene-therapy (experimental scheme as in Fig. 6b). (**e**) Summary of the rectification index of the AMPAR-mediated EPSCs in DH neurons from control (non-inflamed) and CFA-inflamed groups after gene-therapy (experimental scheme of gene-therapy as in Fig. 6b). The rectification index was calculated as above . All data are mean ± SEM. *P < 0.05, **p < 0.01, n.s., non-significant *vs.* control (non-inflamed) group or as indicated.

Finally, for the assessment that antinociception produced by the spinal PKCα inhibition relates to changes in the PKCα-dependent AMPAR trafficking at the DH synapses, we recorded the AMPAR-mediated EPSCs evoked by primary afferent stimulation in DH interneurons. The recorded currents were solely mediated by AMPAR activation since were entirely eliminated by AMPAR antagonists, NBQX (20 μM) and GYKI 52466 (100 μM) (Fig. 8a).

We established that, in consistence with our previous results^5^, the CFA-induced peripheral hypersensitivity led to upregulation of postsynaptic CP-AMPARs at the DH synapses between primary afferents and sensory interneurons. This was demonstrated by the inwardly rectified AMPAR-mediated eEPSCs at positive membrane potentials when recorded in DH interneurons 1 d post-CFA (Fig. 8b,c): the average RI dropped from 0.29 ± 0.04 (n = 20) in control to 0.18 ± 0.02 (n = 12) 1 d post-CFA (*p* < 0.01; Fig. 8b). Pharmacological inhibition of spinal PKC in rats with the developed peripheral pain hypersensitivity completely abolished that rectification 1 d post-CFA when recorded in DH interneurons at 3 to 5 h after i.t. injection of 39 μM chelerythrine. The average RI was 0.25 ± 0.03 (n = 12) in DH interneurons from CFA-inflamed rats treated with chelerythrine, demonstrating a great similarity with that value in control (non-inflamed animals, *p* = 0.44 compared to control; Fig. 8b,c). Having such effect in CFA-inflamed animals, chelerythrine influenced neither RI nor *I–V* shape of the AMPAR-mediated EPSCs in the DH neurons from non-inflamed animals (the average RI: 0.27 ± 0.02, n = 17 in non-inflamed, chelerythrine-treated group and 0.29 ± 0.04, n = 20, *p* = 0.5 in non-inflamed group without chelerythrine, control; Supplementary Fig.S2). The latter indicates no effect of chelerythrine *per se* on the postsynaptic AMPARs at the DH synapses (wherein no inflammation).

Genetic targeting of the spinal PKCα arrested the inflammatory upregulation of CP-AMPARs at the DH synapses between primary afferents and sensory interneurons. Analysis of the AMPAR-mediated EPSCs recorded in DH interneurons from the AS ODN-and MS ODN-treated CFA-inflamed animals (4 days of gene-therapy) revealed that the average RI 1 d post-CFA was: 0.18 ± 0.02 (n = 6) for the MS ODN-treated (a decrease by ~62%, *p* < 0.01 compared with non-inflamed group; Fig. 8e) but 0.25 ± 0.02 (n = 11) for the AS ODN-treated animals (*p* <0.05 compared with the MS ODN-treated, CFA-inflamed group and *p* = 0.5 compared with non-inflamed group; Fig. 8e). Altogether this indicates that antinociception produced by targeting the spinal PKCα (pharmacological or genetic inhibition) is causally linked to the PKCα-dependent inflammatory upregulation of postsynaptic CP-AMPARs at the DH synapses between primary afferents and sensory interneurons and, hence, peripheral nociceptive hypersensitivity in persistent inflammatory conditions.

## Discussion

Despite the latest improvements in pain medication emerged from fundamental and clinical studies, treating persistent pain remains quite challenging. For effective treatment of persistent pain it appears  that the development of new approaches is required, aimed specifically at targeting the molecular mechanisms to provide benefits of preventing (or at least minimizing) side effects, commonly associated with the repeatedly injected existing drug medications.

Fundamental studies of the central mechanisms underlying chronic pain unveiled that CP-AMPARs play a crucial role in maintaining of persistent inflammatory pain^2,3,5,13^. This role originates from the activity-dependent trafficking of AMPARs, which balance has been broken in the DH interneurons in chronic pain conditions^5,7,8,12^. As a result, upregulated CP-AMPARs (both synaptic and extrasynaptic receptors) cause the hyperexcitability of DH interneurons and hyperexcitation of the DH circuitry, linked to pain chronification in central pathways^9^. Our recent study demonstrated that inhibition of spinal CP-AMPARs with the activity-dependent antagonists produced a marked alleviation of inflammatory pain at the periphery without any detectable side effects^10^. Cumulatively, it argues that a decline in upregulation of spinal CP-AMPARs would represent a promising strategy for pain treatment in preventing the long lasting pain. Based on our previous studies of the molecular mechanism of inflammatory pain development and maintenance, a mechanism-based treatment was utilized in this study, which focuses on preventing upregulation of CP-AMPARs through inhibiting an upstream trigger of such upregulation in central pain pathways, namely PKCα^5,8^. We probed two different strategies for targeting the spinal PKCα – pharmacological inhibition or gene-silencing – through local delivery of a drug (genetic material) into the lumbar spinal cord. To build-up the therapeutic profile of such targeting, we designed and examined different experimental schemes, pre- and post-treatment of peripheral inflammatory hypersensitivity, for  thorough testing of any effects emerging acutely (within hours) and/or with a delay following treatment (over days) by measuring the nociceptive threshold in animals twice an hour for 5 to 6-h time window and then each day for over the next few days. The time-courses that we built up for pain relief have demonstrated a high potency of PKC antagonists in relieving thermal inflammatory pain following spinal administration. A broad, cell-permeable PKC antagonist chelerythrine effectively alleviated peripheral inflammatory pain when used as either pre- or post-treatment, whose therapeutic effects were detectable even at nanomolar concentrations (330 nM, 660 nM), the range close to IC_50_ for PKC inhibition (established as 660 nM). The antinociceptive effects of chelerythrine were time-dependent – pain relief to last for few days required higher drug concentrations (at the micromolar range). High potency of chelerythrine for reliving inflammatory pain via spinal administration is consistent with the alleviation of inflammatory hyperalgesia by chelerythrine injections  into the hypothalamus^14^, one of the brain structures for pain modulation, and through intracerebroventricular injections^15^. Importantly that in addition to its antinociceptive effect, chelerythrine recovered locomotive deficit in CFA-inflamed animals; moreover, it declined anxiety, a common sign of painful states. The latter is consistent with the PKC involvement in memory formation and anxiety status^16^ and suggests a complex link between locomotion, anxiety-like behaviour and nociceptive processing in central pathways^17^. Our behavioral studies provide for the first time evidence of the antinociceptive effect produced by PKCα (C2–4) inhibitor peptide. The effects of C2–4 peptide were tested out for different peptide concentrations administered i.t. after the peripheral hypersensitivity had been developed (post-treatment). Although PKCα (C2–4) inhibitor peptide produced the antinociceptive effects at high potency (10 μM) and being emerged rapidly (within half an hour), its effect was markedly lower than that produced by chelerythrine (Fig. 7c). This represents the next line in support for the role of other PKC isoforms in maintaining of persistent pain of inflammatory origin. The PKC enzyme family includes at present at least twelve isoforms, few of those were recognized as being particularly involved in pain. In the first instance, PKCβ and PKCγ were both upregulated in inflammation^18–20^, thereby, implicated into the development of CFA-induced hypersensitivities^18,20,^ while atypical spinal PKCs (e.g. PKMζ) were reported to undergo activation in both development and maintenance of pain states^21^. Apart from the inflammatory pain, a bulk of experimental studies on rodents demonstrated that PKC isoforms, especially gamma subtype^22^, play the important role in allodynia and hyperalgesia of different etiology^23–26^. Our results of gene-silencing of spinal PKCα have confirmed the therapeutic potential of acute genetic inhibition (knockdown) of PKCα in the treatment of persistent inflammatory pain. A downregulation of the PKCα protein expression in the DH of the spinal cord after spinal administration of AS ODN specific for PKCα has been confirmed by Western blot analysis, in full compliance with a use of such approach in our earlier studies^8^. The antinociceptive effects of gene-therapy, confirmed for either pre- or post-treatment of persistent inflammatory pain, were the most promising in shortening the pain maintenance.

Numerous signalling cascades are coupled to PKCα, hence, its inhibition can trigger modifications of various proteins. Nevertheless, our patch-clamp recordings of the AMPAR-mediated EPSCs in DH interneurons evoked by primary afferent stimulation clearly stated that pharmacological inhibition of spinal PKC with chelerythrine declined the inward rectification of postsynaptic AMPAR-mediated currents at positive membrane potentials in DH neurons from CFA-inflamed animals (under persistent inflammatory conditions). Given that the inwardly rectified currents featured CP-AMPARs^5,6,13^, this indicates a decline in inflammatory upregulation of the postsynaptic CP-AMPARs following inhibition of spinal PKC *in vivo*. As a functional outcome, we have observed alleviation of inflammatory pain hypersensitivity at the periphery. Similar ablation of the upregulated CP-AMPARs at the DH synapses between primary afferents and DH interneurons was found following gene-therapy (gene-silencing of spinal PKCα). These findings further argue towards a causal link between pain relief at the periphery and arrested upregulation of the postsynaptic CP-AMPARs at DH synapses by targeting the spinal PKCα. The molecular mechanism of PKCα-dependent upregulation of CP-AMPARs includes the activation of PKCα under painful conditions as an upstream trigger for the AMPAR protein phosphorylation, a subsequent disrupting of the receptor binding to its synaptic anchoring protein, followed by receptor’s internalization^5^. Multiple sites for the PKC-mediated phosphorylation of AMPAR subunits have been distinguished: serine residue S880 for GluA2^5^, S831^27^, S818, and S816 for GluA1^28–30^ that opens considerations for multiple involvement of different PKC enzyme isoforms in both inflammatory pain development and maintenance.

Summarizing, the PKCα-dependent upregulation of CP-AMPARs in DH interneurons is causally linked to the persistent inflammatory pain hypersensitivity at the periphery. Targeting the spinal PKCα, either through pharmacological inhibition or gene-silencing, produced relief in persistent inflammatory pain via ablation of the inflammatory-induced upregulation of postsynaptic AMPARs at the DH synapses between primary afferents and sensory interneurons. This treatment, mechanism-based on targeting the spinal PKCα, will minimize a risk of damage to interneurons not involved in nociceptive processing, thus, reducing side effects on the cellular and systemic levels as one being tailored to nociceptive interneurons only. Such strategy may open a new route  towards cutting-edge approaches to treat chronic pain states through targeting the mechanism-based chronification in central pain pathways.

## Methods

### Animals

Animals were 18- to 30-day-old male Wistar rats. All animal procedures were approved by the Animal Ethics Committee in Bogomoletz Institute of Physiology (Kyiv, Ukraine) and were in accordance with the European Commission Directive (86/609/EEC) and ethical guidelines of the International Association for the Study of Pain. The efforts were made to minimize animal suffering and to reduce the number of animals used.

### Intrathecal catheter implantation

For local delivery of a drug into the spinal cord, a catheter was implanted into the lumbar spinal segment, as we have described in details earlier^10,11^. Briefly, after a rat was anesthetized with a mixture of ketamine (70 mg kg^–1^) and xylazine (15 mg kg^–1^), a catheter (PE-10) was inserted into the subarachnoid space at the rostral level of the spinal cord lumbar region through incision at the atlanto-occipital membrane, using a stereotaxic frame system. Animals received postoperatively bicillin (0.6 million U kg^–1^) and dexamethazon (60 mg kg^–1^). Catheterized animals were taken into experiments after complete healing of surgical incision (typically 3 to 5 days postoperatively). The position of the catheter was confirmed for each animal after termination of behavioural studies or prior to the spinal cord preparation.

### Induction of peripheral inflammation

Unilateral peripheral inflammation was induced in rats by an intraplantar injection of complete Freund’s adjuvant (CFA, 50 µl of an oil-saline (1:1) emulsion) given subcutaneously into one hind paw as described previously^7,9,10^. Saline (0.9%) injection was used as a control.

### Intrathecal drug administration

For pharmacological inhibition, a potent inhibitor of PKC chelerythrine chloride and PKCalpha (C2–4) inhibitor peptide were used. An animal received a single i.t. injection of a drug (10 μl) given at various concentrations. For i.t. injections, a 25-gauge needle connected to a 25 μl Hamilton syringe were both used. Injection of a drug was followed by administration of saline to flush the catheter.

For gene-silencing approach we used the antisense (AS) oligodeoxynucleotides (ODN) specific to PKCα with the following sequence: 5′-GACATCCCTTTCCCCCTCGG-3′. Missense (MS) oligodeoxynucleotides with the sequence of 5′-CGTCCTCAGTCGTCCCTCAC-3′ were used as a control. Animals received a daily injection of AS ODN or MS ODN (10 μg/10 μl) as indicated in the text.

### Western blot analysis

The PKCα protein expression level was determined in the spinal cord of the rats injected with AS- or MS ODNs (n = 6/group). The tissue from the dorsal spinal cord was dissected out and immediately frozen in liquid nitrogen. The tissue was then homogenized in an ice-cold RIPA-buffer (1:3) that contained 20 mM Tris–HCl, 150 mM NaCl, 1 mM EDTA, 1% NP-40, 1% sodium deoxycholate, 0.1% SDS, 1 µM leupeptin and 1 mM protease inhibitor PMSF (pH 7.5). The homogenate was centrifuged (15 min at 11,000×*g*, 4 °C) and the supernatant was collected. After measuring the protein concentration, the protein fraction was separated using 10% polyacrylamide gel with 0.1% SDS and then electrophoretically transferred onto a nitrocellulose membrane (90 min at 200 mA). For the Western blot analysis human/mouse/rat affinity purified polyclonal antibody for PKCα (1:1000, incubation for overnight, 4°С, CAF5340-SP, R&D Systems Inc, UK) and monoclonal mouse primary antibody for β-actin (1:1000, 2 h incubation, A1978, Sigma, USA) were used. The proteins were detected with anti-goat or anti-mouse secondary antibodies and visualized with chemiluminescence reagents provided with the ECL kit (Amersham Pharmacia Biotech, Piscataway, NJ) and exposured to film. The PKCα protein level, normalized to the corresponding β-actin value, was calculated for each individual sample as the relative expression (to naïve group).

### Hargreaves plantar test for the thermal nociceptive threshold measurement

Behavioural testing was performed to measure the peripheral nociceptive threshold  to the thermal (heat) stimulus in rats, using the Hargreaves technique, as we have described previously^10,12^. Briefly, after an animal habituated to the Plexiglas chamber located above a light box (Ugo Basile Model 7370 Plantar Test), a radiant heat was applied to the middle of the plantar surface of one hind paw. The light beam was automatically turned off when animal lifted its paw. The trial was repeated 3–5 times with an interval between measurements 3 to 5 minutes. The time between the start of stimulus and animal lifted its paw, the withdrawal latency, was measured, which represents the thermal nociceptive threshold.

### Open-field test for the assessment of animal locomotion and anxiety

The open-field test was performed to assess the locomotive behaviour of the animals before and following experimental treatment, as described in details in our previous studies^10,11^. Briefly, a tested animal was placed in the open-field arena representing a 75 × 75 × 40 cm wooden box with a digital camera (Logitech C270) attached above the arena to record the animal relocations. The total distance travelled by an animal for the defined period of time (5 minutes) was calculated.

Anxiety is a commonly used readout of side effects that could rise following treatment. Since animal anxiety is characterized by a reduced exploratory behaviour – animals avoid entering an open area (centre of the arena) by keeping close to the walls and corners (closed area) – for the assessment of anxiety, we analysed the number of crossings by animals the centre of the arena. Testing was performed in a quiet room by the same experimenter carrying out a given test in a blind to drug treatment condition manner.

### Spinal cord slice preparation

Spinal cord slices of the lumbar spinal cord region (L_4–5_) were prepared as described previously^8,9^. Briefly, the spinal cord was quickly removed and placed in an ice-cold dissection solution that contained (in mM) 250 sucrose, 2 KCl, 1.2 NaH_2_PO_4_, 0.5 CaCl_2_, 7 MgCl_2_, 26 NaHCO_3_, 11 glucose, oxygenated with 95% O_2_ and 5% CO_2_. Transverse slices (350-μm thick) with attached dorsal roots (8–15 mm) were cut with an HA 752 vibratome (Campden Instruments, Loughborough, UK). Slices were maintained at room temperature in a physiological Krebs bicarbonate solution that contained (in mM) 125 NaCl, 2.5 KCl, 1.25 NaH_2_PO_4_, 2 CaCl_2_, 1 MgCl_2_, 26 NaHCO_3_, 10 glucose, oxygenated with 95% O_2_ and 5% CO_2_ (pH 7.4).

### Electrophysiology

Whole-cell recordings of the AMPAR-mediated EPSCs were made from DH interneurons of the superficial dorsal horn (lamina I-II), using an Axopatch 200B amplifier controlled with pClamp 9.2 software (Molecular Devices, USA). Neurons were visualized with an infrared optics using a ×60.09 water-immersion objective on an Olympus BX50WI upright microscope (Olympus, Japan). Patch pipettes had the resistance of 5–6.5 MΩ when filled with an internal solution containing (in mM) 130 Cs-methylsulfonate, 10 NaCl, 10 EGTA, 2 CaCl2, 10 HEPES, 5 QX-314, 0.1 spermine tetrahydrochloride, 2 Mg-ATP, and 0.1 Na-GTP (pH 7.2). The dorsal root (afferent fibres) was stimulated with a suction electrode by applying the current pulses of an increased stimulus intensity (70 to 400 μA, 0.1-ms-duration) at a low frequency (0.1 Hz). Only stable EPSCs were selected if they displayed the same latency (the interval between the stimulus artefact and the evoked current). The recordings (3 to 5 trials) were averaged and the peak current amplitude was estimated for each tested neuron. The membrane resistance was constantly monitored by applying  a short hyperpolarizing pulse (–5 mV). To isolate the AMPAR-mediated component, EPSCs were recorded in the continuous presence of APV (50 μM), bicuculline methiodide (10 μM), and strychnine hydrochloride (2 μM). For the *I-V* relationship, the membrane potential was held from –70 mV to +50 mV (in a 10- to 20-mV increment). For the *I-V* rectification of the AMPAR-mediated EPSCs, the rectification index (RI) was calculated as the peak current amplitude at +40 mV to that peak amplitude at –70 mV.

### Statistical analysis

All data are presented as mean ± SEM with *n* referring to the number of animals tested or the number of cells recorded. For behavioural studies, a statistical difference was analysed using one-way or two-way analysis of variance (ANOVA) followed by Bonferroni *post hoc* test where appropriate. For electrophysiological recordings and Western blot analysis Student’s t-test (two-tailed unpaired) was used. A *P* value of less than 0.05 was considered as statistically significant for either test.

### Experimental drugs

CFA was purchased from Sigma Chemical Co. (St. Louis, MO, USA), chelerythrine chloride – from Tocris Bioscience (Ellisville, MO). AS ODN and MS ODN were purchased from ISIS Pharmaceuticals Inc. (Carlsbad, CA) or Sigma-Aldrich (UK).

## Electronic supplementary material

Supplementary Dataset
