# Supplementary material for: Spinal PKCα inhibition and gene-silencing for pain relief: AMPAR trafficking at the synapses between primary afferents and sensory interneurons
Source: Sci Rep. 2018 Jul 6;8:10285. doi: 10.1038/s41598-018-28512-9 (PMC6035211; doi:10.1038/s41598-018-28512-9)
Supplement: Supplementary file 1 — Supplementary Dataset [file 41598_2018_28512_MOESM1_ESM.doc]

**SupplementaRY information**

to the article

**“Spinal PKCα inhibition and gene-silencing for pain relief: AMPAR trafficking at the synapses between primary afferents and sensory interneurons”**

by Olga Kopach, Volodymyr Krotov, **Angela Shysh, Andrij Sotnic, Viacheslav Viatchenko-Karpinski, Victor Dosenko,** Nana Voitenko

**
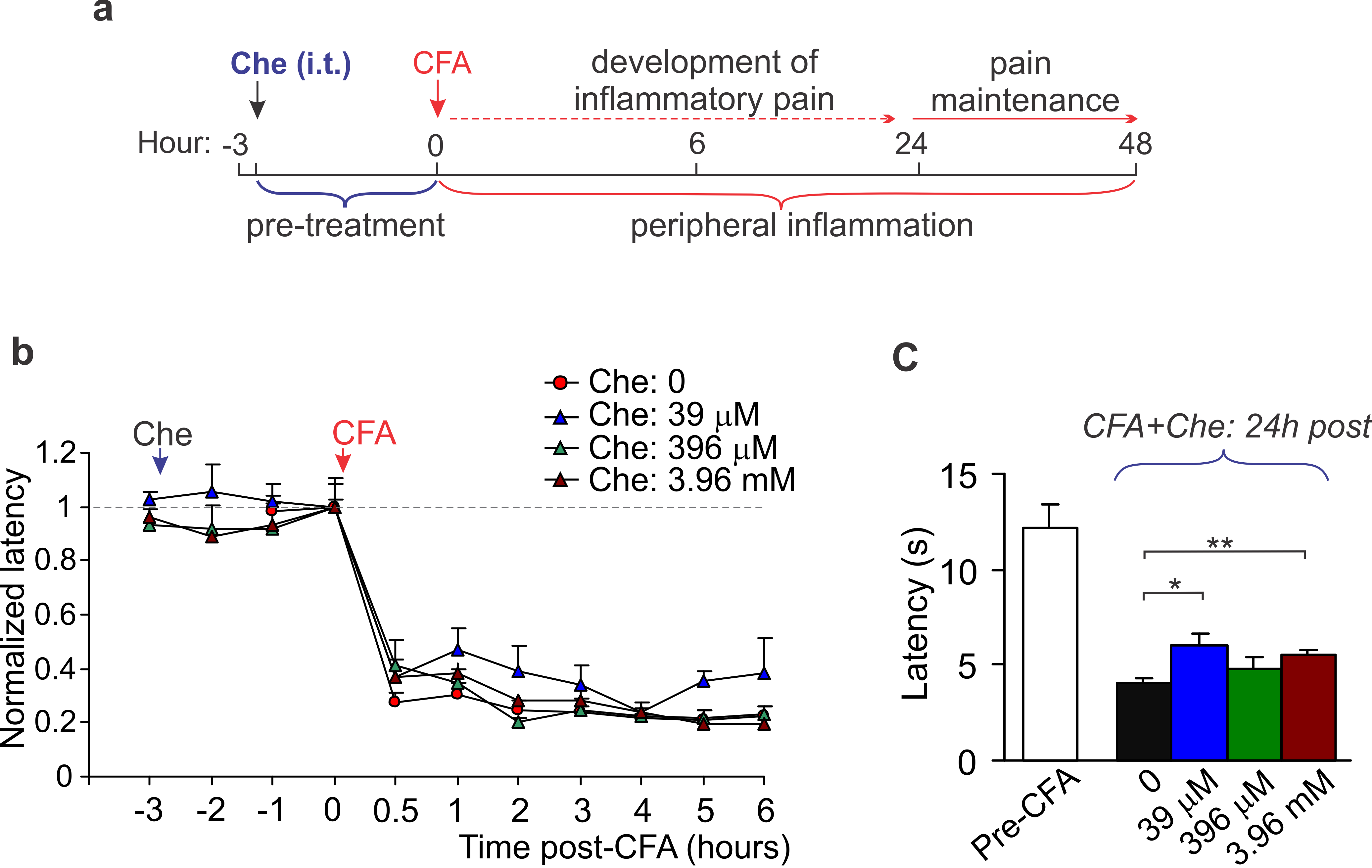
**

**Figure S1.** Pharmacological inhibition of spinal PKC with chelerythrine produced the antinociceptive effects on inflammation-induced peripheral nociceptive hypersensitivity in rats depending on the time of initiating a pre-treatment.

1. A cartoon depicting the experimental scheme of pre-treatment with chelerythrine (Che, intrathecal, i.t., administration 10 μl/rat ) given before the induction of peripheral inflammation with an intraplantar injection of CFA 3 h in advance.
2. The time course of changes in the thermal nociceptive threshold in the CFA-inflamed rats those received pre-treatment with different concentrations of chelerythrine. The threshold is the paw withdrawal latency normalized to the time of CFA injection (“0” time; induction of inflammation).
3. Summary of changes in the thermal nociceptive threshold (the paw withdrawal latency) in CFA-inflamed animals on the day 1 post-CFA following pre-treatment with chelerythrine at different concentrations.

Data are shown as mean ± SEM. **P* < 0.05, **p < 0.01.


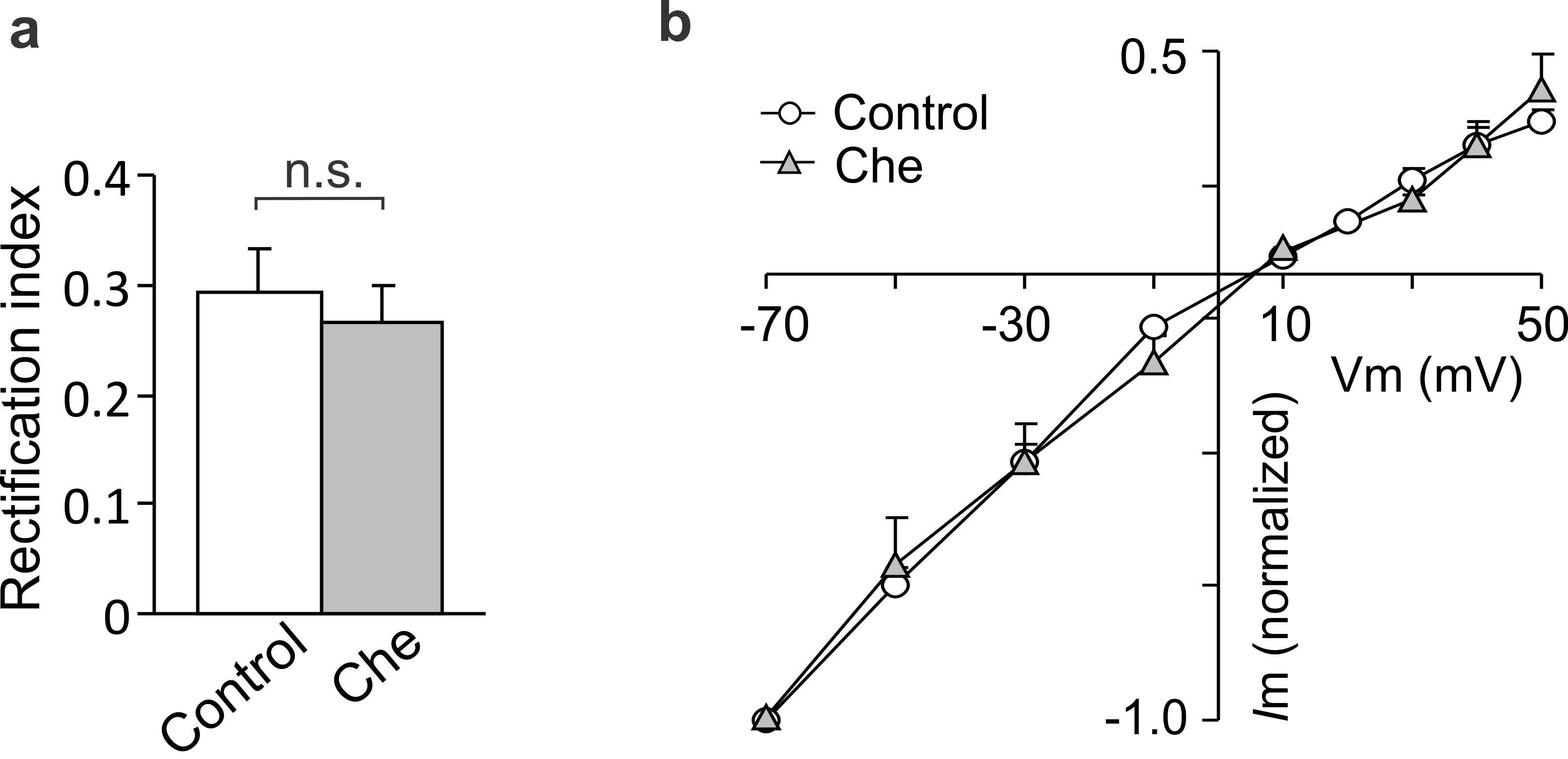


**Figure S2.** Pharmacological inhibition of spinal PKC *in vivo* does not influence the postsynaptic CP-AMPARs at the DH synapses between primary afferents and sensory interneurons in control (non-inflamed) conditions.

**(a–b)** Summary of the rectification index of the AMPAR-mediated EPSCs (**a**) and their *I-V* relationship (**b**) in DH neurons from control (non-inflamed) animals given treatment with 39 μM chelerythrine (Che, intrathecal, i.t., administration; experimental scheme as in Fig. 3a). The rectification index was calculated as the ratio of the current amplitude at +40 mV to that at –70 mV (n = 20 neurons tested in control group, n = 17 neurons tested at 3 to 5 h after chelerythrine administration).

All data are mean ± SEM; n.s., non-significant.
